# Factors Impacting Quality of Life in a Group of Iranian Patients in Chronic Oral Mucosal Disease Following Treatment?

**DOI:** 10.1002/cre2.922

**Published:** 2024-08-28

**Authors:** Mohammad Shooriabi, Sedigheh Modarres Mousavy, Farideh Kaabomeir, Elham Jafari

**Affiliations:** ^1^ Department of Oral Medicine, School of Dentistry Ahvaz Jundishapur University of Medical Sciences Ahvaz Iran; ^2^ Department of Knowledge and Information Science Shahid Chamran University of Ahvaz Ahvaz Iran; ^3^ School of Dentistry Ahvaz Jundishapur University of Medical Sciences Ahvaz Iran

**Keywords:** chronic disease, Iranian, lichen planus, mouth diseases, oral medicine, oral ulcer

## Abstract

**Objective:**

The objective of this study was to investigate the impact of treatment on the quality of life of patients with chronic oral mucosal diseases. Specifically, the study aimed to determine how treatment affects the changes in the quality of life of these patients.

**Methods:**

This descriptive study involved 220 patients diagnosed with chronic oral mucosal lesions. Data were collected using the Chronic Oral Mucosal Disease Questionnaire, validated for use in Persian/Farsi. The study population was selected through convenience sampling. Data analysis employed descriptive statistical methods, including frequency and percentage distribution tables, graphs, measures of central tendency, and dispersion. Additionally, confidence intervals were utilized for the studied ratios and indices.

**Results:**

Among the study population, 129 (58.6%) were male and 91 (41.4%) were female. The mean quality of life, as assessed by the utilized questionnaire, was 61.9 ± 13.2. The results indicated that females and unemployed individuals reported lower quality of life compared to males and employed individuals, respectively, with a statistically significant difference (*p* < 0.05).

**Conclusion:**

In the population studied the quality of life of patients with chronic mucous membrane diseases was influenced by various factors, including gender, income, employment, and place of residence.

## 1 Introduction

Chronic mucosal diseases encompass a range of autoimmune, inflammatory, and infectious conditions that impact the soft tissue of the mouth. These ailments carry substantial physical, social, and psychological implications for the patient. Among the most prevalent chronic oral mucosal diseases are oral lichen planus, pemphigus vulgaris, and recurrent aphthous stomatitis (Burket, Greenberg, and Glick 2021).

However, oral lichen planus is a chronic disease characterized by periods of remission and relapse, necessitating long‐term symptomatic treatment and extended follow‐up examinations due to its potentially malignant nature. The primary treatment for the disease involves the use of topical corticosteroids, although complete cure is often not achieved (Raj and Raj 2023).

Pemphigus vulgaris is a life‐threatening autoimmune disorder that results in the formation of blisters and ulcers on the skin and mucous membranes, including the oral mucosa. Chronic ulcerative lesions are associated with the disease, and oral disease specialists play a crucial role in diagnosis and treatment. Patients often require prolonged use of immunosuppressive drugs under physician supervision to manage the disease (Costan et al. 2021).

Recurrent aphthous stomatitis is a common condition affecting the oral mucosa, leading to the development of one or more painful ulcers in the mouth that typically heal on their own within 7 to 14 days. In some cases, the frequency of ulcer occurrence is so high that the patient's mouth may never be ulcer‐free. Treatment options vary based on the severity of the lesions and often involve the use of systemic or topical corticosteroids (Sridevi Anjuga and Aravindha 2020).

Diseases can cause discomfort and pain while eating and speaking, as well as hinder compliance with oral hygiene practices like brushing and flossing. These issues can negatively impact the patient's personal and familial relationships, ultimately reducing their quality of life (Mumcu, Hayran, and Ozalp 2007; Radwan‐Oczko et al. 2018). The World Health Organization defines health as encompassing physical, mental, and social well‐being, rather than just the absence of disease. Therefore, evaluating therapeutic interventions and disease indicators should also consider the patient's quality of life during treatment, highlighting the importance of human values (Larsen 2022; King and dan Pemela 2012). Most scientists agree that the concept of quality of life encompasses five dimensions: physical, mental, social, spiritual, and symptoms related to disease or treatment changes. These dimensions directly impact a person's happiness and satisfaction, serving as key indicators of quality of life (King and dan Pemela 2012). General questionnaires are commonly used to measure quality of life, offering acceptable validity and reliability. However, they may not capture the specific clinical manifestations of different diseases. In dentistry, the Oral Health Impact Profile‐14 and 36‐Item Short‐Form Health Survey are widely used general questionnaires. On the other hand, special questionnaires are tailored to specific disease categories, offering higher sensitivity and responsiveness, albeit with less comprehensive coverage. An example of a disease‐specific questionnaire is the Chronic Oral Mucosal Disease Questionnaire (COMDQ), designed for diseases affecting the oral mucosa. The COMDQ provides valuable insights into the quality of life of individuals affected by such diseases, demonstrating acceptable validity and reliability (Rajan et al. 2014; Liu et al. 2012).

In two Iranian studies conducted individual studies using the Persian COMDQ questionnaire to assess the quality of life of patients with pemphigus vulgaris, oral lichen planus, and recurrent aphthous stomatitis. Their findings indicated that, as evaluated through this questionnaire, the quality of life of patients was moderate to good (Saberi, Tabesh, and Darvish 2022; Lavaee et al. 2019). In 2021, an Indonesia study demonstrated that sociodemographic characteristics such as employment and gender influenced the quality of life in patients with mental illness (Shafie, Samari, and Jeyagurunathan 2021).

Therefore, this study aimed to evaluate the quality of life in patients undergoing treatment for chronic mucous membrane diseases, including oral lichen planus, pemphigus vulgaris, and recurrent aphthous stomatitis. The evaluation will consider various factors such as place of residence, employment, income, and gender, with the goal of understanding their impact on the patients' quality of life.

## 2 Material and Methods

### 2.1 Study Population

Upon receiving approval from the ethics committee (Ahvaz Jundishapur University of Medical Sciences: IR.AJUMS.REC.1400.038), a total of 220 eligible patients suffering from pemphigus vulgaris, oral lichen planus, or recurrent aphthous stomatitis and seeking treatment at three private centers were included in the study from March 2021 to December 2022. Patients expressed their informed consent to participate in the study, and a convenience sampling method was utilized. Two private clinics specializing in oral diseases in Fars province and one private practice in Khuzestan province, selected based on the number of specialists in the respective provinces, served as patient referral centers. Before obtaining patient consent, a comprehensive explanation of the research, its objectives, confidentiality, and voluntary participation was provided.

### 2.2 Inclusion and Exclusion Criteria

The inclusion criteria for the study were as follows (Burket, Greenberg, and Glick 2021; Wiriyakijja et al. 2020):

— Patients aged 18 years and older.
— Confirmation of disease diagnosis based on clinical history and histological evaluation.
— Diagnosis of oral lichen planus in accordance with the diagnostic criteria of the World Health Organization (WHO).
— Confirmation of pemphigus vulgaris diagnosis using the direct immunofluorescence test.
— Undergoing treatment for more than 14 days based on the treating physician's recommendation.

Exclusion criteria included:

— Presence of oral ulcers with blood test indications of anemia.
— Evidence of dysplasia in biopsy samples.
— Symptoms suggest sensitivity to dental materials.
— Symptoms suggest the presence of leukoplakia lesions resulting from systemic lupus erythematosus and GVHD.
— Symptoms of chronic orofacial neuropathic pain.
— Presence of a serious underlying medical condition with an ASA classification of 3 or higher.

Inability to read and comprehend the questionnaire.

### 2.3 Research Workflow

The Persian/Farsi version of COMDQ, which was confirmed for validity and reliability (Shirzad et al. 2018), was administered to the patients in a paper‐based format. They were then requested to complete it. This questionnaire comprises 26 questions that assess four dimensions of the patient's quality of life: pain, functional limitation, medication and treatment, and mental and emotional status. The questions are scored on a 5‐point Likert scale, ranging from “most of the time” (Score 4) to “never” (Score 0). Consequently, the total score for all questions in the questionnaire falls within the range of 0 to 104. Patients with higher scores exhibit a lower quality of life. The raw scores were converted into percentages, and each patient's quality of life was categorized based on the obtained score. Individuals with a score of 0% to 25% were classified as having an excellent quality of life, those with a score of 26% to 50% as having a good quality of life, those with a score of 51% to 75% as having a moderate quality of life, and those with a score of 76% to 100% as having a poor quality of life (Shirzad et al. 2018; Rajan et al. 2014).

To align with the study's objectives, a section was incorporated into the questionnaire to gather information on patients' age, gender, employment status, education level, income, and place of residence.

### 2.4 Statistical Analysis

The data were analyzed using descriptive statistical methods, such as frequency and percentage distribution tables, graphs, measures of central tendency, and measures of dispersion. Additionally, confidence intervals were calculated for the studied ratios and indices. The simultaneous effect between the studied variables was examined using linear regression. The analysis was conducted using SPSS software, and the significance level for all the aforementioned tests was set at 0.05. Due to the normal distribution of the data, parametric tests (such as regression analysis) were utilized.

### 2.5 Calculation of Sample Size

Based on the research's objectives, the authors' perspective, and previous studies (Rajan et al. 2014), and considering *α* = 0.05, *s* = 19.73, and *d* = 2.6, the sample size was determined to be 221 individuals using the following formula:

$$n=\frac{{\left(Z_{1-\frac{\alpha }{2}}\right)}^{2}\left(s^{2}\right)}{{\left(d\right)}^{2}}.$$

## 3 Results

A total of 220 patients participated in the current study, with 129 (58.6%) being male and 91 (41.4%) female. The mean age of the participants was 41.8 ± 12.7 years. Among the participants, 119 individuals (54%) had lichen planus, 86 (39%) had recurrent aphthous stomatitis, and 15 (6.8%) had pemphigus vulgaris. Additional demographic characteristics of the study population are presented in Table 1. Furthermore, 172 individuals (78.2%) were employed, while 48 (21.8%) were unemployed. In terms of education, 82 individuals (37%) had less than a high school diploma, 46 (20%) held such a diploma, and 92 (41%) had degrees higher than a high school diploma. Only 16 patients (7.3%) reported a monthly income exceeding 60 million Rials (more than 200 dollars). Forty‐two participants (19%) were residents of Khuzestan, with the remainder residing in Fars province. The mean quality of life, as assessed by the questionnaire, was 61.9 ± 13.2, indicating a moderate level of quality of life. Table 1 displays the scores of the participants in each dimension, while Table 2 presents the evaluation results of the quality of life based on demographic variables. The findings suggest that women exhibited a lower quality of life than men, and unemployed individuals reported a lower quality of life compared to employed individuals. Multivariate analysis, utilizing a linear regression model to explore the simultaneous effect of demographic variables and the dependent variable of quality of life, revealed that female gender, unemployed status, and Khuzestan residency were each associated with a lower quality of life (Table 3).

**Table 1:** Total scores on COMDQ.

	Mean	Minimum	Maximum	SD
Pain and functional limitation	67.8	50.0	88.9	14.4
Drug and treatment	59.5	45.8	95.8	17.6
Social and emotional	61.2	46.4	92.9	15.5
Patient support	53.3	12.5	68.8	11.1
Total	61.9	49.0	82.7	13.2

**Table 2:** Evaluation of the mean of quality of life according to demographic variables.

Variable	Mean pain and functional limitation (SD)	Mean medication and treatment (SD)	Mean social and emotional (SD)	Mean patient support (SD)	Mean overall QOL (SD)
Gender					
Male	66.6 (14.3)	57.5 (16.8)	58.7 (15.6)	49.8 (11.7)	59.8 (12.2)
Female	69.4 (14.5)	62.3 (18.4)	64.8 (14.6)	58.4 (8.0)	64.8 (14.0)
Employment					
Employed	66.2 (13.8)	55.7 (15.0)	57.8 (13.1)	52.1 (11.3)	59.4 (11.4)
Unemployed	73.4 (15.4)	73.1 (19.6)	73.5 (17.2)	57.8 (9.4)	70.9 (14.9)
Disease type					
OLP	74.8 (12.1)	69.4 (18.1)	70.5 (14.5)	57.7 (9.6)	69.8 (12.4)
RAS	54.8 (5.8)	46.7 (6.0)	48.5 (6.5)	52.3 (49)	50.9 (4.6)
Pemphigus	86.1 (0.0)	54.2 (0.0)	60.7 (0.0)	25.0 (0.0)	62.5 (0.0)
Education					
Lesser than diploma	76.7 (9.3)	64.2 (16.4)	68.3 (13.8)	48.2 (12.4)	67.2 (10.1)
Diploma	60.2 (6.1)	49.9 (13.4)	49.1 (9.0)	51.5 (4.9)	53.5 (8.3)
Higher than diploma	63.6 (16.9)	60.1 (18.7)	61.0 (15.7)	58.8 (9.7)	61.4 (15.2)
Income/month					
Less than $100	68.1 (14.3)	58.0 (16.4)	60.5 (15.4)	52.3 (10.5)	61.3 (12.6)
$100–$200	73.4 (12.5)	76.5 (18.8)	73.7 (14.5)	63.8 (9.2)	72.7 (13.8)
More than $200	57.6 (13.7)	56.2 (20.1)	55.3 (10.1)	53.5 (14.1)	56.1 (12.6)
Location					
Khuzestan province	71.7 (16.9)	65.7 (17.9)	66.8 (15.6)	57.4 (13.7)	69.2 (14.9)
Fars province	66.6 (13.6)	58.0 (17.2)	59.9 (15.2)	52.4 (10.3)	60.6 (12.4)

**Table 3:** Simultaneous effect of demographic variables and self‐assessment of patients (independent variables) with total questionnaire scores (dependent variable) using a linear regression model.

Variable	*B* (regression coefficient)	*p* value
Age	−0.053	0.296
Gender	3.612	0.007
Employment	19.371	0.0001
Disease	2.848	0.108
income	−3.903	0.006
Education	−1.139	0.150
Location	5.054	0.030

## 4 Discussion

The study's findings indicate that individuals with chronic oral mucosal diseases, despite undergoing treatment, experienced only moderate quality of life scores across all dimensions of the CMODQ questionnaire. Furthermore, their quality of life was significantly influenced by gender, employment status, and place of residence.

Previous research has suggested a higher prevalence of oral lichen planus, recurrent oral aphthous, and pemphigus vulgaris in females compared to males (Raj and Raj 2023). However, our study found a higher number of chronic oral mucosal diseases in males, aligning with the findings of two studies in India and Lebanon (Bhatnagar et al. 2013; EL Toum et al. 2018). Discrepancies in the results may be attributed to differences in the populations studied. Our study focused on individuals referred to a physician due to the severity of oral mucosal problems, while a study of the South Indian population (Omal et al. 2012) included all individuals visiting a research site for dental treatments.

The results reveal that despite receiving treatment, the oral health‐related quality of life of the participants was at a moderate level, with most patients reporting poor scores in the areas of pain and movement limitation. Specifically, they encountered significant difficulties with eating sour, fried, or dry foods, drinking hot or cold beverages, maintaining oral health and hygiene, and using artificial teeth. These findings are consistent with those of a study in India (Rajan et al. 2014).

The findings of this study are consistent with findings from a study in southern England, which utilized the Oral Health‐Related Quality of Life (OHR‐QoL) questionnaire to evaluate the quality of life of patients with oral diseases (Llewellyn, Johnson, and Warnakulasuriya 2004). Two approaches can be considered to explain these results. First, based on the findings of a study (Mumcu, Hayran, and Ozalp 2007) that reported that patients with active oral ulcers experience a lower quality of life than those without ulcers, it can be inferred that the management of oral lesions in the participants of this study was inadequate. This may have resulted in the failure to heal the ulcers and address the physical problems of the oral mucosa, leading to a moderate level of oral health‐related quality of life for the patients. Another approach involves taking a more comprehensive view of the definition of oral health‐related quality of life. In addition to physical health, this approach considers the mental, social, and functional aspects of the patients' health. Therefore, the objective of treatment should not solely focus on symptom elimination but also on addressing the mental, psychological, and social dimensions of the patient (King and dan Pemela 2012). In this context, a study on patients with chronic oral mucosal diseases by an Iranian researcher revealed that most patients were fearful of transmitting the disease to their families and expressed concerns about the unpredictable course of the lesions in the future. Additionally, all patients expressed a desire for more information about their disease (Nassab et al. 2021). It appears that the treatment team pays more attention to the physical symptoms of the patients rather than addressing the factors that could enhance their quality of life.

The study's findings revealed that females have a lower quality of life than males, consistent with previous studies (Lavaee et al. 2019; Rajan et al. 2014). This could be attributed to a lower threshold for pain tolerance, physical disability, and higher mental stress in females (Vadivelu et al. 2017; Graves et al. 2021). One study demonstrated that females have less pain tolerance than males (Vadivelu et al. 2017), while another study showed that females experience higher levels of stress than males (Graves et al. 2021). Based on these results, it can be inferred that incorporating sedative drugs into the treatment regimen of female patients may help improve their quality of life, although further studies are required to confirm this.

The study also indicated that unemployed patients (those with an income of less than 60 million Rials per month) have a lower quality of life compared to other patients with chronic oral mucosal diseases. In a study on Swedish adults, Norström et al. (2019) demonstrated that unemployment and low income can reduce quality of life, increase stress, and have negative impacts on health, supporting the results of the present study. However, it is important to consider the specific characteristics of the studied population, as research has shown that the effects of unemployment on health can vary across different societies and populations (Wilson and Walker 1993). These findings are consistent with other studies that have investigated the effects of unemployment on public health (Johansson, Böckerman, and Lundqvist 2020; Stauder 2019). Notably, no study evaluating the impact of income and employment on the quality of life of patients with chronic oral mucosal diseases was found in the literature, making the present study the first to address this issue.

Two separate studies reported that people with low education have a lower oral health‐related quality of life than other patients (Tsakos 2008; Taghavi Bayat, Huggare, and Akrami 2019). This result was not consistent with the results of the present study. In this study, patients with a high school diploma had a better quality of life compared to patients with a lower or higher degree of education. This inconsistency may be related to differences in the questionnaire type and the difference in the items related to oral health. However, the small number of patients with high‐school diploma education (20.9%) in the present study compared to patients with lower education (37.3%) or higher education (41.8%) may also be due to the lack of effect of education level on the quality of life.

The present study revealed that patients living in Khuzestan have a lower quality of life than patients living in Fars province, which is in line with the results of a study (Slade 2012) that investigated the oral health‐related quality of life in six different population groups, such as urban versus nonurban communities, people living in big versus small cities of Canada, African American versus Caucasian American people. The results of his study revealed that cultural differences have an independent effect on a person's reaction to oral diseases in people with teeth.

Some of the limitations of this study included coexistence of other systemic diseases in some patients, the nonuniformity of the progression of disease in different patients, and the nonuniformity of the severity of oral lesions in different patients, as all these may affect the quality of life of patients.

## 5 Conclusion

In the population studied, the quality of life of patients with chronic mucous membrane diseases is impacted by a range of factors, such as gender, income, employment, and place of residence. It is crucial for the treating team to take these factors into consideration and address them to enhance the overall well‐being of these patients.

## Author Contributions

Mohammad Shooriabi contributed to the study conception and design, as well as data gathering. Sedigheh Modarres Mousavy also contributed to the study conception and design. Farideh Kabomir provided input and contributed to the study conception and design and data analysis. Elham Jafari contributed to both the study conception and design, as well as data gathering.

## Conflicts of Interest

The authors declare no conflicts of interest.

## Data Availability

All data generated or analyzed during this study are included in this published article.
